# Reviewing the Impact of Gluten-Free Diets on Child and Adolescent Psychiatric Disorders

**DOI:** 10.7759/cureus.114662

**Published:** 2026-08-17

**Authors:** Hannah E Stokan, Arya Gajwani, Charlena Melnyk

**Affiliations:** 1 Psychiatry and Behavioral Sciences, The University of Texas Medical Branch at Galveston, Galveston, USA

**Keywords:** autism and gut-brain axis, childhood-onset schizophrenia, complementary and alternative medicines, gluten-free diet, non-celiac gluten sensitivity

## Abstract

Gluten-free diets (GFDs) are increasingly used by families as a complementary intervention for pediatric psychiatric disorders despite limited evidence supporting their efficacy. Proposed mechanisms include immune-mediated inflammation, altered gut-brain signaling, and increased intestinal permeability that may influence neuropsychiatric function. This review aimed to systematically evaluate the evidence for GFD use in children and adolescents with psychiatric disorders without diagnosed celiac disease.

A structured literature search was conducted using Ovid Medical Literature Analysis and Retrieval System Online (MEDLINE), PsycINFO, and ClinicalTrials.gov to identify English-language studies published between January 2000 and August 2024. Studies involving participants ≤20 years old with a psychiatric disorder who underwent a GFD intervention for at least six weeks were reviewed. Studies involving diagnosed celiac disease, acute medical illness, or non-gluten dietary interventions were excluded. Study characteristics, intervention details, and psychiatric outcomes were extracted and qualitatively synthesized.

Four studies met inclusion criteria, including three studies in autism spectrum disorder (ASD) and one case report in schizophrenia. Across ASD studies, evidence was mixed. Two randomized controlled trials found no significant improvement in core ASD symptoms compared with control diets, although one reported significant reductions in gastrointestinal symptoms and modest behavioral improvements. A prospective observational study reported numerical improvements in gastrointestinal complaints, caregiver-reported behavior, and autism-related symptom scores in a subset of participants; however, changes in autism symptom scores did not reach statistical significance. The schizophrenia case report described substantial improvement and eventual resolution of psychotic symptoms following initiation of a GFD in an adolescent with non-celiac gluten sensitivity. No eligible studies were identified for attention-deficit/hyperactivity disorder (ADHD), anxiety disorders, obsessive-compulsive disorder (OCD), mood disorders, disruptive behavior disorders, Tourette syndrome, post-traumatic stress disorder (PTSD), or substance use disorders. Overall, the available evidence was limited by small sample sizes, short intervention periods, methodological heterogeneity, and moderate-to-high risk of bias.

Current evidence supporting GFD use in pediatric psychiatric disorders is sparse and heterogeneous. While some children may experience improvements in gastrointestinal symptoms and associated behavioral difficulties, there is insufficient evidence to support routine implementation of a GFD for core psychiatric symptoms. The near absence of studies outside ASD highlights a significant gap in the literature. Larger, well-designed randomized controlled trials are needed to clarify efficacy, identify potential biological responders, and determine whether subsets of children with gluten sensitivity or immune dysregulation may derive meaningful benefit from dietary gluten restriction.

## Introduction and background

Children with psychiatric disorders are often treated with psychotropic medications along with educational and behavioral interventions. However, an increasing number of parents are turning to Complementary and Alternative Medicine, particularly the gluten-free diet (GFD), for symptom relief [1]. Gluten, a protein complex found mainly in wheat and composed of gliadin and glutenin, is widely used in both staple and processed foods [2]. Gliadin is believed to be a key driver of gluten-related inflammation and gastrointestinal symptoms. Gluten-related disorders encompass distinct clinical entities, including autoimmune celiac disease, IgE-mediated wheat allergy, and non-celiac gluten sensitivity. Unlike celiac disease, which is characterized by autoimmune enteropathy, or wheat allergy, which involves an allergic immune response, non-celiac gluten sensitivity is defined by intestinal and/or extraintestinal symptoms triggered by gluten ingestion in the absence of celiac disease or a wheat allergy. Although proposed mechanisms include innate immune activation and altered intestinal permeability, the pathophysiology of non-celiac gluten sensitivity remains incompletely understood. Inflammatory immune responses as such can lead to increased intestinal permeability (“leaky gut”), where pro-inflammatory cytokines and neurotransmitters can enter systemic circulation and cross the blood-brain barrier, affecting the neurochemistry of the brain [3].

The link between gluten and psychiatric disorders, particularly schizophrenia, was first explored after World War II, when areas with lower grain consumption saw fewer hospital admissions for schizophrenia [4]. Studies from the early 1960s, including a 1973 follow-up, suggested that patients on a GFD improved faster and were discharged sooner than those consuming wheat, a concept coined by Dr. Francis Curtis Dohan as "The Cereal Theory" [5-7]. Although early investigations focused primarily on schizophrenia, interest in GFDs has expanded substantially over the past two decades, particularly within the autism spectrum disorder (ASD) community. Families of children with ASD increasingly pursue dietary interventions in an effort to improve gastrointestinal symptoms, behavioral concerns, and core autism-related features despite limited and inconsistent evidence supporting their efficacy.

While research on the GFD’s effectiveness is still in its early stages, a 2019 study found that 72% of families relied on healthcare providers for diet information, and 33% did not disclose supplement use due to concerns about physician knowledge or judgment [1]. Given the increased interest in gluten, it is important for clinicians to have knowledge about the current evidence base on the use of GFD. This review examines the current evidence regarding the use of GFD in children and adolescents with psychiatric disorders without diagnosed celiac disease.

This article was previously presented as a meeting abstract at the 2025 American Psychological Association annual conference from August 7-9. 

## Review

A systematic literature search using predefined eligibility criteria was performed. This review was not prospectively registered. An electronic literature review was conducted using Ovid Medical Literature Analysis and Retrieval System Online (MEDLINE), PsycINFO, and ClinicalTrials.gov databases. Search terms included “Diet, Gluten Free”, “Gluten, Gliadin intolerance, sensitivity, restriction”, “preschool child (2 to 5 years)”, “child (6 to 12 years)”, “adolescent (13 to 20 years)”, “Elimination Diet”, “Celiac Disease, sprue”, “wheat hypersensitivity, allergy”. Two independent reviewers screened and collected data according to previously decided study criteria. Articles were included if they assessed the use of GFDs as a treatment modality for at least six weeks in participants aged 20 or younger. The eligible studies were limited to English-language, peer-reviewed publications between 2000 and August 2024. 

The literature review included case reports, case series, retrospective studies, observational studies, longitudinal studies, open trials, randomized controlled clinical trials, meta-analyses, and systematic reviews. The search was further refined by targeting mental health treatments for the following disorders: “generalized anxiety,” “separation anxiety,” “social anxiety,” “anxiety,” “adhd,” “autism spectrum disorder (ASD),” “autism,” “obsessive compulsive disorder (OCD),” “ocd,” “posttraumatic stress disorder (PTSD),” “major depressive disorder (MDD),” “bipolar disorder,” “disruptive mood dysregulation disorder (DMDD),” “oppositional defiant disorder (ODD),” “conduct disorder,” “Tourette syndrome,” “reactive attachment disorder,” “panic disorder,” “schizophrenia,” and “substance use”. Studies with participants who had diagnosed celiac disease, acute illnesses, or assessed non-gluten dietary factors were excluded to focus specifically on the effects of a GFD. The study search strategy is summarized in Table 1, with detailed inclusion and exclusion criteria outlined in Table 2.

**Table 1:** Electronic Literature Search Strategy MEDLINE: Medical Literature Analysis and Retrieval System Online

Category	Description
Databases Searched	Ovid MEDLINE, PsycINFO, ClinicalTrials.gov
Search Terms	"Diet, Gluten Free"; "Gluten"; "Gliadin"; "Gluten intolerance"; "Gluten sensitivity"; "Gluten restriction"; "Elimination Diet"; "Celiac Disease"; "Sprue"; "Wheat hypersensitivity"; "Wheat allergy"; "Preschool child (2–5 years)"; "Child (6–12 years)"; "Adolescent (13–20 years)"
Screening Process	Two independent reviewers screened studies and extracted data according to predefined eligibility criteria.
Inclusion Criteria	Participants ≤20 years of age; gluten-free diet intervention for ≥6 weeks; English-language; peer-reviewed studies; published between 2000–August 2024.
Exclusion Criteria	Studies not meeting the predefined eligibility criteria (see Table 2 for complete inclusion and exclusion criteria).

**Table 2:** Inclusion and Exclusion Criteria ADHD: attention-deficit/hyperactivity disorder; ASD: autism spectrum disorder; Bipolar Disorder: bipolar disorder; DMDD: disruptive mood dysregulation disorder; MDD: major depressive disorder; OCD: obsessive-compulsive disorder; ODD: oppositional defiant disorder; PTSD: post-traumatic stress disorder; GFD: gluten-free diet

	Inclusion Criteria	Exclusion Criteria
Population	Children and adolescents ≤20 years of age with a psychiatric disorder (generalized anxiety, separation anxiety, social anxiety, anxiety disorders, ADHD, ASD, OCD, PTSD, MDD, bipolar disorder, DMDD, ODD, conduct disorder, Tourette syndrome, reactive attachment disorder, panic disorder, schizophrenia, substance use disorders)	Participants with diagnosed celiac disease or acute medical illness
Interventions	GFD	Dietary interventions not specifically evaluating gluten restriction (e.g., other elimination diets or nutritional interventions)
Comparisons	Standard diet, control group, placebo, baseline/pre-post comparison, or no comparison (for observational studies and case reports)	None
Outcomes	Psychiatric symptoms assessed using standardized psychiatric rating scales or validated clinical outcome measures	Studies not reporting psychiatric outcomes following GFD
Timing	Minimum intervention duration of six weeks	Intervention duration <6 weeks
Settings	English-language, peer-reviewed studies published between January 2000 and August 2024	Non-English publications
Study Design	Case reports, case series, retrospective studies, observational studies, longitudinal studies, open-label trials, randomized controlled trials, systematic reviews, and meta-analyses	Editorials, commentaries, conference abstracts without full manuscripts, and studies lacking original clinical data

Systematic searches of MEDLINE and PsycINFO databases yielded 170 and 312 articles, respectively. Following screening for adherence to predefined inclusion criteria, two articles from MEDLINE and two from PsycINFO were selected for detailed analysis. A search of Clinicaltrials.gov did not yield any relevant results.

Of the articles reviewed, nine involving patients with co-existing celiac disease were excluded; these studies addressed a variety of psychiatric diagnoses, including ASD, attention-deficit/hyperactivity disorder (ADHD), major depressive disorder (MDD), and obsessive-compulsive disorder (OCD). The final selection comprised three studies on ASD and one on schizophrenia; these are depicted in Table 3. 

**Table 3:** Summary of the Included Studies ASD: autism spectrum disorder; ATEC: Autism Treatment Evaluation Checklist; GFD: gluten-free diet

Psychiatric Condition	Sample Size	Age of Participants	Time on GFD Diet	Control (No Diet Change)	Outcome Measures	Authors
ASD	37	2-12 years	6 months	No	ATEC score, behavior, digestive symptoms	Stoieva et al. [7]
ASD	66	36-69 months	8 weeks	No	Autistic symptoms, maladaptive behavior, intellectual abilities	Piwowarczyk et al. [8]
ASD	80	4-16 years	6 weeks	Yes	Rome III questionnaire, Gilliam Autism Rating Scale-2	Ghalichi et al. [9]
Schizophrenia	1	16 years	2 years	No	Neuroinflammation	Eaton et al. [10]

Evidence for improvement in autism-related behavioral traits was limited. In a prospective observational study, 37 children aged two to 12 years with ASD eliminated gluten from their diet for six months [7]. The Autism Treatment Evaluation Checklist (ATEC) was used to assess psycho-cognitive effects [11]. After six months, ATEC scores decreased numerically from 61 to 50-60 points; however, this change was not statistically significant (p>0.05). Caregivers reported behavioral improvement in 30% of participants, stool improvement in 20%, and resolution of gastrointestinal complaints in 40%. Additionally, 27% of participants had elevated IgG antigliadin antibodies and tissue transglutaminase IgA, suggesting possible underlying gluten-related immune activity.

Additionally, a randomized, controlled, single-blinded trial assessed the effects of a GFD in 66 children aged 36-69 months with ASD over six months [8]. After an eight-week GFD run-in period, the participants were randomized into two groups: a gluten-free or a gluten-containing diet, and they continued this for six months. Children with wheat allergies and celiac disease were excluded. No significant differences in ASD core symptoms were observed between groups, as measured by the Autism Diagnostic Observation Schedule (ADOS-2) [12]. However, both groups showed notable improvements from baseline to the six-month follow-up in restrictive and repetitive behaviors, social communication, and overall autism symptoms, as reported by parents and assessed through additional standardized scales, including the Social Communication Questionnaire (SCQ), Autism Spectrum Rating Scales (ASRS), and Vineland Adaptive Behavior Scales, Second Edition (VABS-2) [13-15]. Observation of changes in both gluten and gluten-free groups suggests that these changes were not attributable to the dietary intervention itself.

In a third randomized controlled trial, 80 children with ASD were assigned to either a GFD (GFD, n = 40) or a regular diet (RD, n = 40) for 6 weeks [9]. Gastrointestinal symptoms were assessed using the Rome III Diagnostic Criteria questionnaire and the Gilliam Autism Rating Scale 2 questionnaire (GARS-2) for assessing psychometric properties [16, 17]. The GFD group showed a significant decrease in the prevalence of GI symptoms (p < 0.05, 40.57% to 17.10%), whereas the RD group showed an insignificant increase (42.45% to 44.05%). GFD intervention also resulted in a significant decrease in behavioral disorders (80.03±14.07 to 75.82±15.37, p < 0.05), while the RD group had a non-significant increase (79.92 to 80.92).

A case report describes a young male who first experienced intermittent auditory and visual hallucinations at age eight [10]. By age 15, he was hospitalized and diagnosed with MDD with paranoid features. Treatment with escitalopram (10 mg/day) and aripiprazole (5 mg/day) proved ineffective, leading to two additional hospitalizations within months. At age 16, he was hospitalized with command hallucinations, homicidal ideation, and self-harm, resulting in a new diagnosis of schizophrenia with paranoid features and treatment with venlafaxine (150 mg/day) and risperidone (1.5 mg/day). During this admission, elevated IgE antigluten antibodies indicated non-celiac gluten sensitivity. After initiation of a GFD during hospitalization, the patient's psychiatric symptoms improved over the course of his admission; however, interpretation is limited by concurrent psychiatric treatment and medication management. Over the next two years on a GFD, he remained symptom-free, gradually tapered off risperidone, and eventually discontinued it entirely. Because dietary modification occurred alongside ongoing psychiatric treatment, the independent contribution of GFD cannot be determined.

No studies were found to evaluate a GFD for the treatment of children and adolescents for ADHD, MDD, OCD, generalized anxiety disorder, separation anxiety, social anxiety, post-traumatic stress disorder (PTSD), bipolar disorder, disruptive mood dysregulation disorder, oppositional defiant disorder, conduct disorder, Tourette syndrome, reactive attachment disorder, panic disorder, or substance use disorders.

This review found limited, heterogeneous evidence supporting the use of a GFD in children and adolescents with psychiatric disorders. Across the included studies, primarily small, randomized trials, findings were mixed. For ASD, a GFD did not consistently improve core diagnostic features but showed modest benefits in gastrointestinal symptoms and some behavioral domains. Evidence in psychiatric disorders outside ASD was extremely limited, consisting of a single case report in schizophrenia. As such, these findings should be considered hypothesis-generating rather than evidence of efficacy. Importantly, the highest-quality evidence available consisted of small randomized controlled trials in ASD, whereas evidence for other psychiatric disorders was limited to observational data and a single case report.

A strength of this review is its focused evaluation of GFD in non-celiac pediatric psychiatric populations across multiple diagnoses, incorporating diverse study designs in an underexplored area. However, conclusions are limited by the small number of studies, heterogeneity in design, and reliance on subjective outcomes. Another limitation is the inherent difficulty in isolating the effects of gluten elimination from other concurrent dietary or lifestyle changes that may accompany adoption of a GFD. As a result, any observed clinical improvements cannot be attributed solely to gluten restriction with certainty.

Findings were heterogeneous across studies, reflecting differences in study design, outcome measures, and domains assessed. The use of diverse clinician-administered assessments, caregiver-reported questionnaires, gastrointestinal symptom measures, and laboratory biomarkers further limits direct comparison across studies and precludes interpretation of similar findings as replication of a common treatment effect. Studies excluding children with celiac disease or wheat allergy showed no significant benefit in core symptoms, contrasting with more consistent improvements reported in celiac populations. Variability in participant selection, adherence, intervention duration, and outcome measures likely contributes to differing results.

One possible explanation for the observed improvements is that some participants may have had unrecognized gluten-related disorders, such as celiac disease or non-celiac gluten sensitivity, although this remains speculative because these conditions were not consistently evaluated across studies. A previous report by Rubenstein et al. found that one in five preschool-aged children with ASD had used a GFD [18]. Exclusion diets are increasingly common within the ASD community, based on a hypothesized predisposition to increased intestinal permeability, often referred to as “leaky gut” [19, 20]. In the included study by Stoieva et al. [7], 27% of participants had elevated antigliadin and tissue transglutaminase antibodies, suggesting possible underlying immune-mediated pathology. In children with ASD, as noted by Prosperi et al., communication challenges and atypical sensory processing may obscure gastrointestinal symptoms, leading to underdiagnosis and misattribution of behavioral improvements to dietary intervention alone [21].

Given the established association between gluten-related disorders and neuropsychiatric manifestations, clinicians should consider screening for celiac disease or other gluten-related disorders in children with psychiatric symptoms accompanied by gastrointestinal complaints or other clinical features suggestive of gluten-related disease before initiating a GFD. While some families pursue GFD as a complementary approach, clinicians should counsel that evidence for core psychiatric symptoms remains limited. Additionally, practical considerations, including cost, dietary adherence, and potential social impact, should be discussed [22, 23].

Because this review focused specifically on gluten-free dietary interventions, the absence of eligible studies in several psychiatric disorders should be interpreted as reflecting the literature captured by our predefined search strategy and inclusion criteria rather than definitive evidence that no relevant dietary intervention studies exist. Future studies should prioritize large, well-designed randomized controlled trials with standardized outcome measures, longer follow-up periods, and objective biomarkers. While treatment of confirmed celiac disease is associated with improvement in both gastrointestinal and extraintestinal manifestations, including psychiatric symptoms, these findings should not be extrapolated to non-celiac psychiatric populations. Future research should evaluate whether biologically distinct subgroups, including individuals with suspected non-celiac gluten sensitivity, demonstrate differential responses to GFD. Expanding investigation beyond ASD and schizophrenia to other psychiatric disorders may help clarify whether any therapeutic role exists for GFD outside of established gluten-related disorders.

## Conclusions

This review identified a substantial gap in the literature evaluating GFDs in the treatment of psychiatric disorders in children and adolescents without diagnosed celiac disease. Among the four eligible studies, evidence was limited primarily to ASD, with a single case report involving schizophrenia. While some studies reported improvements in gastrointestinal symptoms and select behavioral outcomes, randomized controlled trials did not demonstrate consistent benefits for core ASD symptoms. The available evidence is constrained by small sample sizes, heterogeneous methodologies, short intervention periods, and methodological heterogeneity, limiting the ability to draw firm conclusions regarding efficacy.

At present, there is insufficient evidence to recommend routine implementation of a GFD as a treatment for core psychiatric symptoms in pediatric populations. Evidence outside ASD remains extremely limited and, in the case of schizophrenia, is restricted to a single hypothesis-generating case report. Future research should explore whether clinically distinct subgroups of children with non-celiac psychiatric disorders respond differently to gluten restriction. Clinicians should counsel families on the current limitations of the evidence and consider screening for celiac disease or other gluten-related disorders when clinically indicated, particularly before initiating a GFD. Future research should focus on large, well-designed randomized controlled trials incorporating standardized outcome measures, objective biomarkers, and longer follow-up periods to determine whether specific pediatric psychiatric populations may derive meaningful benefit from gluten restriction.
